# Modern RNA Quantification Methods: From RT-qPCR to Advanced Microscopy

**DOI:** 10.1021/acs.jpcb.5c07484

**Published:** 2026-03-17

**Authors:** Tyrese Boddie, Arianna Lacen, Hui-Ting Lee

**Affiliations:** † Department of Chemistry, University of Alabama at Birmingham, 901 14th Street South, Birmingham, Alabama 35294, United States

## Abstract

RNA plays a crucial role in gene expression, regulation, protein synthesis, and other cellular functions. The diversity that exists between different RNAs makes information beyond their expression level necessary for understanding more about their complex functions in a cell. Conventional ensemble approaches to RNA quantification have been used extensively to measure the quantity of RNA but lack cellular-level spatial information. This review highlights important contributions that high resolution microscopy has made to RNA quantification and cellular biophysics. Using advanced microscopy for precise localization, real-time tracking, and quantitative measurements of RNA increases our understanding of different disease states, cell- and tissue-specific gene regulation, and cellular architecture.

## Graphical abstract

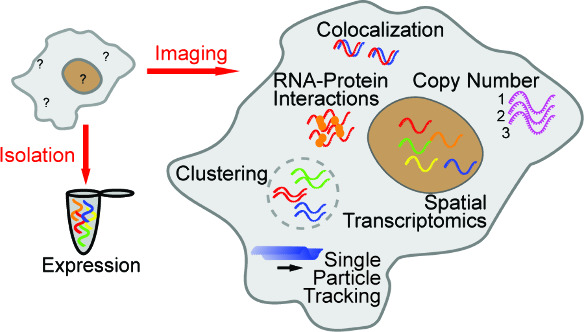

## 1 Introduction

The size, stability, location, function, and amount of RNA in a cellular environment is more diverse than its DNA counterpart.^1^ Isolating RNA from its cellular environment has been a common practice to quantify RNA expression levels and uncover the sequence of unknown RNAs. Conventional quantification of RNA uses methods that isolate RNA from the cell and convert the RNA into complementary DNA (cDNA) to quantify the expression level of RNA.^2,3^ These ensemble methods of RNA quantification measure the average RNA levels across a population of cells and have aided in diagnosing different diseases and established our understanding of RNA biology. However, information provided by these methods is limited to the bulk measurement of RNA expression levels, and quantification of different types of RNA can be challenging. Heterogeneity of RNA expression between neighboring cells is hard to detect by using these conventional methods. Additionally, the spatial and temporal measurement of expressed RNA in single cells cannot be performed solely using cDNA-based techniques.

RNA is relatively unstable and more prone to degradation than DNA due to the abundance of RNases and the ability to self-cleave due to the 2′-OH group. RNA is sensitive to pH, heat, and metal ions. Therefore, the sample processing required to isolate the RNA for quantification can affect the accuracy of quantifying low abundance or unstructured RNAs. The variability in base modifications, structure, length, presence of a poly-A tail, and abundance between different types of RNA provides additional challenges to cDNA-based RNA quantification. For example, transfer RNA (tRNA), ribosomal RNA (rRNA), and some noncoding RNAs (ncRNAs) can form stable secondary structures and possess base modifications.^1,4,5^ These characteristics are functionally important but can cause mismatches or reduce reverse transcription efficiency during cDNA generation and reduce the quantification accuracy.

Simultaneously knowing the expression level and location of RNA in relation to different proteins or subcellular compartments can uncover different interactions and regulatory mechanisms. The use of fluorescent microscopy provides spatial resolution of RNA quantification, which allows for the RNA distribution in the cell to be measured (see recent review articles^6,7^ ). Supplying the spatial resolution with temporal information in live-cell imaging provides velocity and path information on RNA distribution.^7,8^ Recent advancements in super-resolution fluorescence microscopy led to a resolution that was once only capable by using electron microscopy, which cannot be used for RNA quantification. Super-resolution imaging has contributed to the discovery of nanoscale arrangement of RNA and more accurately quantifying RNA copy number in a cell.^9–11^ Some of these imaging techniques have been adopted for diagnostic purposes for quick and sensitive detection of early onset of diseases by using low levels of viral RNA or miRNA as biomarkers,^12,13^ which could not be reliably done using any ensemble method. This reveiw article surveys how advanced microscopy and single-molecule imaging techniques have been used to increase the scope of RNA quantification and overcome challenges of quantifying RNA using ensemble methods.

## 2 Ensemble RNA Quantification

### 2.1 RT-qPCR

Reverse transcription quantitative polymerase chain reaction (RT-qPCR) is the gold standard for quantifying the expression of RNA and is a popular tool for diagnostics.^14–16^ RNA is converted to cDNA using reverse transcriptase, and the cDNA is amplified using PCR. A fluorescent dye or fluorescent-labeled probe binds to the amplified DNA, and the emission is detected after every cycle of PCR. The fluorescence intensity detected during each cycle is proportional to the amount of cDNA amplified. The cycle number when the fluorescent signal surpasses a defined threshold (Ct) is used to calculate the starting amount of cDNA. RT-qPCR can report the relative expression of RNA or the absolute copy number of transcripts. RT-qPCR is a well-established way to quantify RNA, but compared to other methods, it is low-throughput in the amount of RNA that can be quantified at a time. The effectiveness of RT-qPCR is dependent on the efficiency of both reverse transcription and PCR steps.^17^

Viral RNA and eukaryotic RNA that are transcribed from RNA polymerase II (mRNA and some lncRNAs) have poly­(A) tails at the 3′ end. These types of RNAs are easier to quantify using RT-qPCR because poly-dT primers can be used to convert the RNA to cDNA, and poly dT bead pull-down can selectively target poly­(A) tailed RNA. Random sequences or specially designed primers are needed for RNA without a poly-A tail. Using these primers increases the bias and complexity of quantifying RNA without a poly-A tail, and quality control of the primers is needed to reduce/prevent off-target amplification. rRNA makes about 80% ^18,19^ of the total RNA in a cell and needs to be depleted when quantifying other RNA to prevent rRNA dominating the data. However, this additional step of sample processing further increases the chances of RNA degradation. In contrast to rRNA, the amount of small and long ncRNAs (lncRNAs) is very low in the cell. The low copy number, presence of base modifications, and secondary structure formation increase the difficulty of amplifying small RNAs and lncRNAs. Small ncRNAs such as miRNA (21–24 nt) and piRNA (26–31 nt) are of similar length as common primers used for reverse transcription.^20^ Special protocols are needed to generate cDNA from these small ncRNA, but their low copy number, chemical modifications, or secondary structure will still reduce the reverse transcription efficiency. These principles result in the difficulty of distinguishing small ncRNAs from each other or degraded RNA fragments.

#### 2.1.1 Microarray

Microarrays are used to simultaneously quantify multiple RNA through the hybridization of probes with fluorescently labeled cDNA samples in an array chip.^21^ DNA probes are then hybridized to various regions of cDNA using Fluorescence in situ Hybridization (FISH). Fluorescence is detected by scanning the fluorescent signal from each spot after hybridization, which represents a specific gene, and the intensity of the fluorescence is used to measure the abundance of the target gene in the sample.^21,22^ Quantifying RNA using a microarray provides a high-throughput alternative to RT-qPCR by quantifying thousands of transcripts per chip but cannot calculate transcript copy number. Both RT-qPCR and microarray require knowing the target sequence and are unable to reliably detect novel transcripts or unknown splice variants.

#### 2.1.2 RNA Sequencing

RNA sequencing (RNA-seq) measures gene expression either by sequencing a DNA copy of the RNA (cDNA-based RNA sequencing) or by directly sequencing the RNA (direct RNA sequencing). Sequenced fragments of RNA or DNA (reads) are aligned with a reference gene or genome, and abundance measurements are reported by the number of aligned reads. There are well established pipelines for cDNA-based RNA sequencing, and the use of PCR allows for low abundant transcripts to be detected.^3^ cDNA-based sequencing most commonly uses short read platforms (e.g., Illumina) that fragment DNA or cDNA (50–400 bp) before reading, and sequencing is done by synthesis using fluorescently labeled nucleotides and imaging the light emitted.^23,24^ cDNA-based RNA sequencing provides relative expression quantification of RNA. However, cDNA-based RNA sequencing faces the same limitations mentioned in the RT-qPCR section (section 2.1). RNA isoforms or overlapping transcripts are harder to align, therefore harder to quantify when shorter reads are used. Furthermore, any RNA base modifications are lost when converted to cDNA.

In direct RNA sequencing (e.g., Oxford Nanopore), RNA is sequenced directly by translocating through a nanopore protein that is embedded in a synthetic membrane inside of an electrolyte solution, and the changes in current as each base pass through the nanopore are measured to give the identity of the base.^25^ Direct RNA sequencing preserves the information on base modification, so post-transcriptional modifications can be studied. The poly-A tail length and products from alternative splicing are lost during reverse transcription and PCR but can be detected by using direct RNA sequencing. Since RNA is read directly, each read more accurately reflects RNA abundance compared to cDNA-based sequencing.^3,25^ Direct RNA sequencing is essentially a single-molecule technique, since each RNA molecule translocates through the nanopore individually, and the sequence of each molecule is reported. We include it in this section because the individual reads are pooled together for quantification as in the cDNA sequencing methods.

### 2.2 Flow Cytometry and Nano Biopsy

Ensemble techniques like RT-qPCR and NGS use multiple cells and are unable to resolve the RNA expression in individual cells or cell types if gene expression is heterogeneous among cells. Flow cytometry is a cell sorting technique that allows for the analysis of individual cells.^26^ Cells pass through a flow cytometer are sorted based on different characteristics (e.g., size or fluorescence) when passing a sensor. Cells can be sorted by fluorescence using FISH or by cell size, granularity, and other morphological differences based on light refraction. Combining RNA FISH techniques with flow cytometry were used to quantify the expression of different mRNAs and miRNAs.^27–30^ Flow cytometry can also be used in conjunction with NGS techniques for single cell RNA-seq (scRNA-seq). This gives single cell information on RNA expression patterns that were unresolved in bulk RNA-seq.

Even though flow cytometry separates cells, making single cell or cell type RT-qPCR and NGS achievable, the spatial resolution within the cell is lost after the isolation of RNA following flow cytometry. The study of polarized cells, like neurons and endothelial cells, could benefit from technology that quantifies RNA at a sub cellular spatial resolution. Nano biopsies include an array of techniques (atomic force microscopy, nano pipettes, nano straws, and nano tweezers) that can extract RNA and other biomolecules in specific locations of a cell for quantification.^31,32^

## 3 Image-Based RNA Quantification

PCR and sequencing methods for quantifying RNA lack any spatial context, which was overcome by the rise of fluorescence microscopy. Fluorescence microscopy enables spatial quantification of multiple RNAs in relation to different proteins, organelles, or other RNAs, which are traditionally done with diffraction limited microscopy. Diffraction limited microscopy includes wide-field and confocal microscopy, which on their own, can only image samples at a resolution above the diffraction limit of light (approximately 200 nm).^33^ Wide-field microscopy captures emitted light both in and out of the focal plane, which has been used for bulk measurements of RNA abundance and distribution.^34^ Confocal microscopy excludes out of focus and scattered light, allowing measurements of RNA location and abundance in a cell at a higher resolution. This is achieved by a pinhole in the excitation and emission pathways, which excludes out of focus light before it reaches the detector, usually a PMT or EMCCD equivalent.^35^

Fixed-cell imaging has been used to quantify RNA in cells by correlating the fluorescence intensity with the abundance of RNA in a cell at a fixed position in time. Different imaging techniques for RNA in fixed cells aim to increase the signal-to-noise ratio, especially for low expressed RNA, by amplifying the fluorescent signal detected for a target RNA. The high signal-to-noise ratio allows some techniques to be used in live-cell imaging of RNA, which allows for the monitoring of RNA expression and movement in real-time. The fluorescent signal intensity at different spots or regions can be analyzed to give the spatial and temporal distribution of RNA. Many existing analyses used in spatial descriptive statics for geographical study are adapted to this type of analysis.

### 3.1 Fixed-Cell Imaging of RNA

#### 3.1.1 Fluorescence in Situ Hybridization (FISH)

Fluorescence in situ Hybridization (FISH) uses fluorescently labeled complementary oligonucleotides to bind to nucleic acids. The fluorescent oligonucleotides, commonly termed FISH probes, are typically made of either DNA or Protein Nucleic Acids (PNA). These probes are usually single stranded and bind to their complementary target sequence specifically. PNA FISH probes are typically less prone to degradation than DNA probes due to their modified peptide backbone as opposed to the phosphodiester backbone of DNA but are more costly than DNA probes. FISH was originally used to image DNA but was later adapted for RNA.^36^ DNA FISH requires a denaturation step to separate the DNA duplex to allow for probes to hybridize onto a gene of interest.^37^ Since RNA is not in the long polymer double-stranded state, a harsh denaturation step is not required for probes to anneal to RNA. The major challenges of applying FISH to RNA are the lower stability of RNA compared to DNA and the low copy numbers of some RNAs. The low stability of RNA makes the sample preparation process of FISH easily damage the target RNA. Young et al. published a technical review guide for RNA FISH pointing out the general features of gentle sample fixation, mild permeabilization, and the long incubation with FISH probes at a lower temperature (37 °C or room temperature incubation).^38^ The first example of RNA FISH was performed by Singer and Ward to visualize actin mRNA in a culture of chicken skeletal muscle using DNA probes that were 200–300 nt.^36^ They used the fluorescence of a rhodamine-labeled secondary antibody to quantify the copy number of actin mRNA per cell.

#### 3.1.2 smFISH

Single molecule FISH (smFISH) uses multiple short fluorescently labeled oligonucleotide probes (approximately 20 nt long) to target different regions of the same mRNA, which generates a higher signal-to-noise ratio (SNR) than traditional FISH (Figure 1A).^39–41^ The smFISH probes can either have a dye directly conjugated to the oligonucleotide complementary to the target RNA (smFISH) or carry a sequence that is complementary to a specific sequence to anneal to a dye labeled primer (smiFISH). Using multiple fluorescently labeled oligonucleotides to target a single RNA sequence enhances the detection of RNA that is expressed in a low quantity. Having multiple probes bound to the same target sequence increases the signal-to-noise ratio, as the chance of multiple probes nonspecifically binding to the background is lower than a single probe. smFISH has been an important tool for the quantification of mRNAs in different parts of a cell, leading to a better understanding of gene expression in different biological systems. Early examples of smFISH include the technique being used to detect and quantify β-actin mRNA molecules in normal rat kidney cells.^36,41^ smFISH laid the foundation for most of the labeling methods mentioned below, and advancements to smFISH led to the technique being capable of imaging single nucleotide variants (SNVs)^42^ and spliced mRNA.^43^

**Figure 1:**
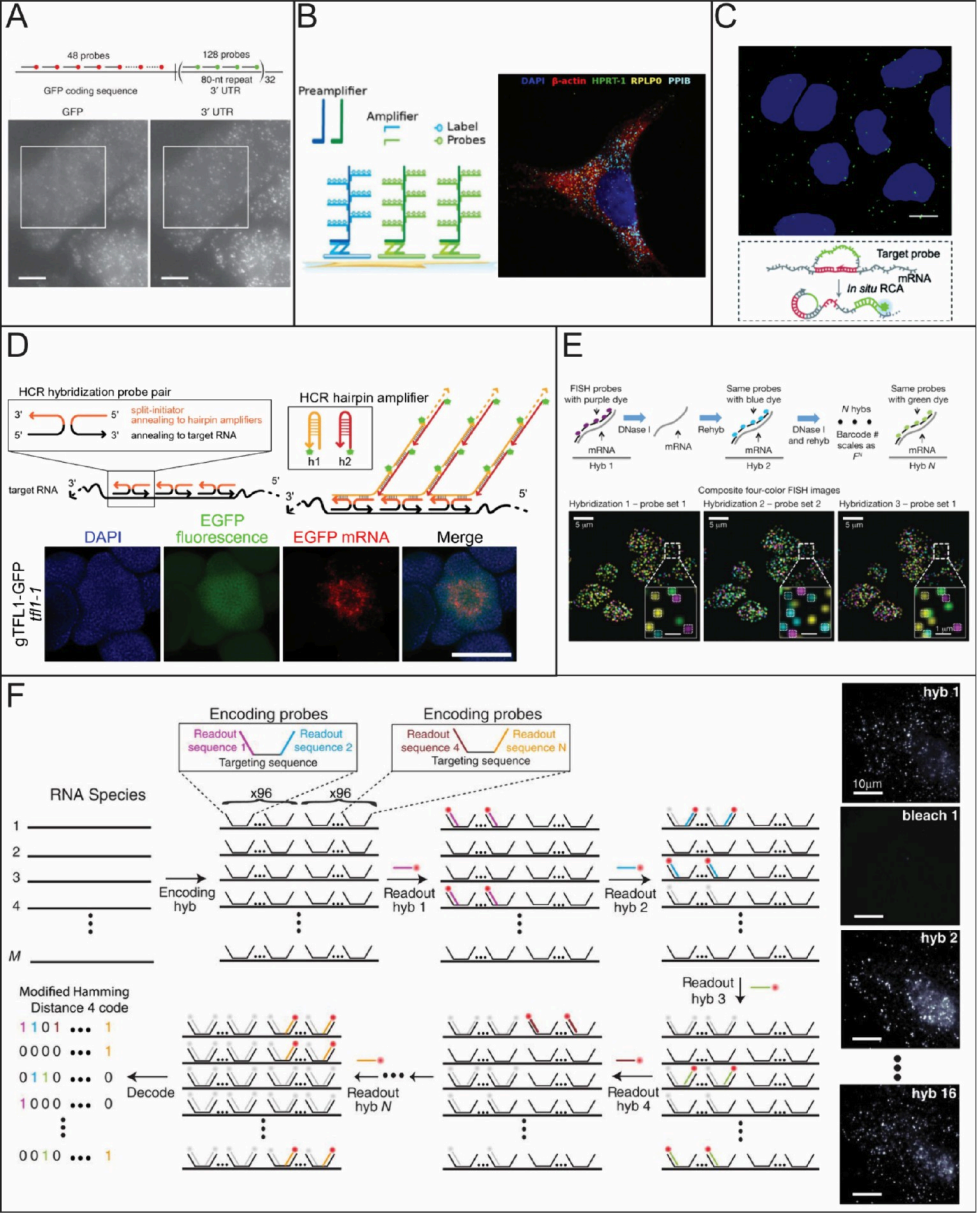
Common fixed-cell imaging techniques used for RNA quantification. (A) Illustration of smFISH (top) to label GFP RNA in CHO cells. Reproduced from ref 39 under Creative Commons License CC-BY-NC-ND. Copyright 2008 Raj A; et al. Published by Springer Nature. (B) Illustration of RNAscope (right) and an example of RNAscope being used for multicolor detection of β-actin, PLP0 (60S acidic ribosomal protein P0), PPIB (peptidylprolyl isomerase B), and HPRT-1 (hypoxanthine phosphoribosyltransferase 1) (left). Reproduced from ref 44 under Creative Commons License CC-BY-NC-ND. Copyright 2012 Wang, F; et al. Published by Elsevier. (C) Illustration of RCAFISH with the target padlock probe (bottom) to image TK1 mRNA in MCF-7 cells. Adapted from ref 50 under Creative Commons License CC BY 3.0. Copyright 2017 Deng, R.; et al. Published by Royal Society of Chemistry. (D) Illustration of HCR FISH (top) and validation of the technique by detecting EGFP mRNA in wild-type *Arabidopsis*. Adapted from ref 46 under Creative Commons License CC BY 4.0. Copyright 2023 Huang, T.; et al. Published by Springer Nature. (E) Principle of seqFISH and example images. Reproduced from ref 54 under Creative Commons License CC-BY-NC-ND. Copyright 2014 Lubeck, E.; et al. Published by Springer Nature. (F) MERFISH workflow (left) and images of RNA molecules in an IMR90 cell after each hybridization round. Adapted with permission from ref 59. Copyright 2015 AAAS.

#### 3.1.3 RNAscope

RNAscope, like smFISH, was developed to increase the SNR of target RNA molecules in a cell or tissue, enabling low expression RNA to be imaged.^44,45^ RNAscope uses a pair of Z-probes for each target sequence (Figure 1B). Each Z-probe is composed of a region complementary to the target RNA sequence and a region complementary to a preamplifier sequence, which is linked by a proprietary spacer sequence. When a pair of Z-probes are bound to the target next to each other, a preamplifier oligo can be hybridized to the Z-probe pairs and makes them form a Z-shape.^44,45^ After hybridization of the preamplifier onto the Z-probe pair, amplifiers containing 20 label probes branch out from the preamplifier. The amplification of fluorescent signal in RNA scope can only occur when Z-probe pairs are hybridized next to each other, so background signal will not be amplified in the instance of nonspecific binding of single Z-probes.^44^

The high specificity of RNAscope was demonstrated in the original publication where 18S rRNA was used as the target.^44^ There was no detection of the target when only one Z-probe was used; however, when double Z-probes were used, the target RNA was able to be detected. RNA scope is also optimized to be used with formalin-fixed, paraffin-embedded (FFPE) methods. These methods are used to preserve tissue samples for histological analysis, but during the process RNA can fragmentize, which makes using smFISH challenging.^44,45^ The smFISH approach to imaging RNA requires multiple probes to anneal to a single RNA molecule for a detectable signal; therefore, short or degraded RNA transcripts would be hard to identify. RNA scope can be used for detecting short or degraded RNA in fixed tissue samples because one RNA molecule is labeled with two probes that bind to multiple fluorescent dyes after amplification. As a quantification tool, RNAscope can only provide relative, semiquantitative information, opposed to smFISH being able to be used for copy number quantification.

#### 3.1.4 HCR-FISH

Hybridization Chain Reaction FISH (HCR-FISH) executes high specific labeling and high SNR through the alternative sequential tethering between two labeled hairpin readout probes (H1 and H2) (Figure 1D).^46,47^ An unlabeled initiator probe is complementary to the target RNA and the first hairpin readout probe (H1). Each labeled readout probe has a 3′ region only specific to the 5′ end of the other labeled readout probe. The hairpin structure and sequence specificity of the readout probes reduce the chance of nonspecific signal accumulation and enhance the SNR.^47^ Upon annealing the initiator probe to the target mRNA, the first readout probe (H1) annealed to the other end of the initiator probe. Annealing of H1 to the initiator probe unfolds the hairpin, leaving a region on H1 available for annealing of H1’s complementary hairpin forming probe, H2. Once H2 anneals to H1, it also unfolds. This allows for the subsequent annealing of multiple H1 and H2 probes. Because of the target specificity of the initiator and hairpin probes, multiple genomic targets can be visualized simultaneously in multiplexing capacity.

Choi et al. utilized HCR-FISH multiplexing capability to simultaneously target five mRNAs in fixed zebrafish embryos.^47^ Given that specificity of HCR-FISH is governed by the initiator probe, multitarget imaging is possible using initiator probes complementary to different H1. The capacity for multiplexing makes HCR-FISH suitable for imaging and quantification such as in tissue samples. One group, Lovely et al., has developed protocols for hybridization and quantification in axolotl tissue samples. Their protocols are based on the third generation of HCR-FISH (HCR v3.0) that utilizes split initiator probes to greatly improve signal-to-noise over previous iterations.^48^ In HCR v3.0 the initiator sequence is cut in half into two probes instead of one probe containing the full initiator sequence as described by Choi et al. in 2014 ^47^ and 2018.^49^ When each half initiator probe binds to its target, the two initiator halves colocalize, allowing for annealing of the first hairpin forming probe and kickstarting HCR.

#### 3.1.5 RCA-FISH

Rolling Circle Amplification FISH (RCA-FISH) is another technique that modifies probe design for higher signal levels.^50^ The main feature of RCA-FISH is the inclusion of a locked nucleic acid padlock probe that is designed to anneal to an RNA target (Figure 1C).^50^ Locked nucleic acids are modified DNA that contain a bridging carbon linking the 2′ oxygen and 4′ carbon of the ribose ring. The purpose of this modification is to lock the ribose into a conformation that enhances the binding affinity to the RNA target.^51,52^ When hybridized to the RNA or cDNA target, the padlock probe has both the 3′ and 5′ ends annealed to the RNA target, leaving the rest of the padlock probe free, forming a loop or bubble-like structure. The two ends of the padlock probe are then ligated, locking the probe to the target. Primers for rolling circle amplification are then annealed to the padlock probe “bubble” and amplification of the padlock probe sequence is carried out by phi29-XT DNA Polymerase, synthesizing multiple copies of the padlock probe. The padlock probe contains a sequence that fluorescent readout probes can hybridize to, allowing for sensitive detection of amplified RCA products; thus, the resultant signal is specific and intense. The intensity of the signal leads to shorter exposure time and increased signal-to-noise. RCA-FISH was used to detect single-base differences between human and mouse β-actin sequences.^50^ The SNR of RCA FISH is dependent on the enzymatic activity of the polymerase.

#### 3.1.6 SeqFISH

Sequential FISH (seqFISH) uses sequential rounds of hybridization and imaging to identify multiple RNA species, utilizing fluorophore-based barcodes. In the early work by Lubeck and Cai,^53^ multiple short (20 nt) probe sets were hybridized to different sections of one mRNA, while the probes in the same set carries the same unique fluorophore. The spatial distribution or spectral colocalization of different color spots generates the specific pattern (barcode) for each RNA. Although they were able to report spatial distribution of a single mRNA molecule to <20 nm resolution, the authors concluded that using the colocalization pattern at the same diffraction-limited spot is more practical and less technically demanding for identifying different RNA species. They used this colocalization pattern to study 32 stress-responsive genes and fixed *Saccharomyces cerevisiae* (budding yeast) cells. The same authors later established seqFISH by modifying the way they utilizing different colors and adding the sequential hybridization process.^54^ In seqFISH, the same set of oligonucleotide probes binding to a specific mRNA species is repeatedly used in multiple rounds of hybridization, but the fluorophore this probe set carries may be different in each round. After each round of hybridization, the sample was imaged, treated with DNase I, and photobleached to remove all of the signals to prepare for the next round. The barcode was read as the order of each color showed up at the same spot (Figure 1E). As long as the probe set of each mRNA species has a unique order of fluorophore color in all rounds, F^N^ RNA species can be identified with N rounds of hybridization with F types of fluorophores or color, e.g., sixteen mRNA species only needed two rounds of hybridization use four fluorophores, 4^2^ = 16. This allows the users to identify high number of RNA species with four fluorophores or less, which has the potential to profile the entire transcriptome at single cell resolution.^55,56^

Profiling transcripts in a single cell using seqFISH is limited by optical resolution, and the density of mRNA as a single transcript is the size of a diffraction-limited spot. To overcome this, the process of seqFISH has been adapted to RNA SPOTs, in which mRNA from cells were extracted and fixed onto an oligo­(dT) surface before multiple rounds of hybridization and imaging.^57^ The RNA immobilization process diluted mRNA to lower the density of RNA under the microscope and allowed better identification of different copies. RNA SPOTs can be considered as a cheaper and more accurate alternative method to transcriptional profiling than cDNA-based RNA-seq, as no polymerase is involved and the oligo­(dT) surface in RNA SPOTs avoids the need for rRNA depletion as in all RNA-seq. Another modified version of seqFISH, seqFISH+, achieved covering more genes in fewer rounds by using primary probes that can bind to a few secondary readout probes, while multiple primary probes bind to a single mRNA species. Using five types of fluorophores and the pseudocolor generated by them, the authors identified 10,000 genes within a single NIH/3T3 fibroblast cell.^58^

#### 3.1.7 MERFISH

Multiplexed error-robust FISH (MERFISH), like seqFISH+, can image thousands of RNA molecules by using encoding probes (Figure 1F).^59,60^ Encoding probes are composed of readout sequences that flank the 5′ and 3′ ends of a target probe sequence. The target probe sequence is complementary to a specific region on an RNA species to be studied. Each encoding probe contains 2 or 3 readout sequences, and each readout sequence is one of the known sequences. Multiple encoding sequences are designed to bind to one RNA target as in smFISH. Multiple runs of fluorescently labeled readout probes are hybridized with the sample sequentially. Each readout probe that binds to a readout sequence is hybridized to the sample, imaged, and photobleached before the next readout probe is introduced. The order of fluorescent signals appearing at each spot can be converted into binary code, thus allowing for subsequent decoding of the RNA at a particular location. (See more details of probe design and decoding instruction in ref 61.)

The earliest demonstration of MERFISH by Chen et al. was used with only a widefield fluorescent microscope.^59^ In the initial report by Chen et al., the group imaged 140 RNA species using a modified hamming distance 4 (MHD4) code that allowed error detection and correction to achieve approximately 80% detection efficiency. To achieve this high detection efficiency, 16 rounds of hybridization were performed.

MERFISH as a tool can image a higher number of total RNA transcripts in comparison to smFISH due to its barcoding approach. The major limitation of MERFISH is the difficulty of identifying different RNA species that have overlapping fluorescent signals due to the resolution limit of the microscope and diffraction limit, which eventually was overcome using expansion microscopy.^61,62^ Jonathan Liu et al. compared MERFISH to both bulk and scRNA-seq in mouse kidney and liver cells to confirm distribution of RNA counts from MERFISH accurately reflected both RNA-seq methods.^63^ MERFISH can detect genes in cells that were undetectable using scRNA-seq, and the total number of transcripts detected by MERFISH surpassed that of bulk RNA-seq. These advantages come from MERFISH using multiple fluorescent probes and rounds of hybridization to increase the SNR. MERFISH has also been optimized to 3D image and spatially profile 100–200 μm thick tissue samples from mouse brain hypothalamus and cortex using spinning disk confocal microscopy.^64^

### 3.2 Live-Cell Imaging of RNA

#### 3.2.1 MS2-MCP System

The MS2-MCP system the is most common technique for live-cell imaging of RNA and has been used in live-cell imaging to track the movement of mRNA and ncRNA.^65^ The MS2- MCP system incorporates 24 copies of RNA stem-loop structures to the 3′UTR of a gene of interest. The stem-loop structures are derived from an MS2 bacteriophage and can be bound by two MS2 coat proteins (MCP) fused to a green fluorescent protein for live-cell imaging. In a recent study,^66^ Chiu et al. used the MS2-MCP system to monitor Influenza A virus (IAV) replication. The stem-loops used in the MS2-MCP system were incorporated into the viral RNA (vRNA) of IAV to track the viral replication over time. Using this approach, the vRNA signal and distribution were able to be monitored for approximately 18 h post infection (hpi). This group revealed that PB2-vMSL (a plasmid reporter containing the PB2 subunit of IAV viral polymerase) replication only occurred in apoptotic cells and IAV replicates asynchronously in apoptotic cells, suggesting that IAV vRNA replication is facilitated through apoptosis.

In 2023, Yucen Hu et al. used an MS2-MCP based approach to monitor gene expression of mRNA in live *C. elegans*.^67^ In comparison to the previously mentioned study and traditional MS2-MCP systems, only 8 MS2 stem-loops were inserted next to the gene of interest because of the low efficiency of inserting the approximately 1300 nt needed for the expression of 24 repeats of MS2 stem-loops. Additionally, many other studies point out the uncertainty of using 24 repeats of MS2 stem-loops in accurately representing endogenous RNA dynamics. This uncertainty comes from the MS2 stem-loops potentially preventing or delaying RNA degradation. Their revised MS2-MCP system, named “MS2-based signal Amplification with Suntag System (MASS)” makes it easier to insert the MS2 stem-loop sequences due to only 8 repeats being needed (Figure 2A). In addition to a shorter number of MS2 repeats, a 24xSunTag array was fused to the MCP. The 24xSunTag was used to bind up to 24 GFP molecules; therefore, fusing this SunTag array to MCP enables the recruitment of up to 384 GFP per 8xMS2 tagged sequences. The efficacy of MASS was demonstrated through the live-cell imaging of ACTB mRNA to monitor the signal-to-noise ratio, velocity, and intensity differences of MASS systems with a different number of SunTag repeats (24x, 12x, and 6x) than the traditional MS2-MCP system. The SNR and average intensity of all MASS variations were higher than the traditional MS2-MCP system, but the velocity of ACTB mRNA decreased as the number of SunTag repeats decreased, with the velocity of 6xSunTags being the most like the velocity of the 24x MS2-MCP system. Following these results, endogenous C42D4.3–8xMS2 mRNA dynamics were monitored in live *C. elegans* after laser wounding. Hu revealed the increased expression of C42D4.3–8xMS2 mRNA after laser wounding determined by the detection of GFP near the wound and monitoring of the movement of GFP signal after wounding and fusion events between GFP loci in real time.

**Figure 2:**
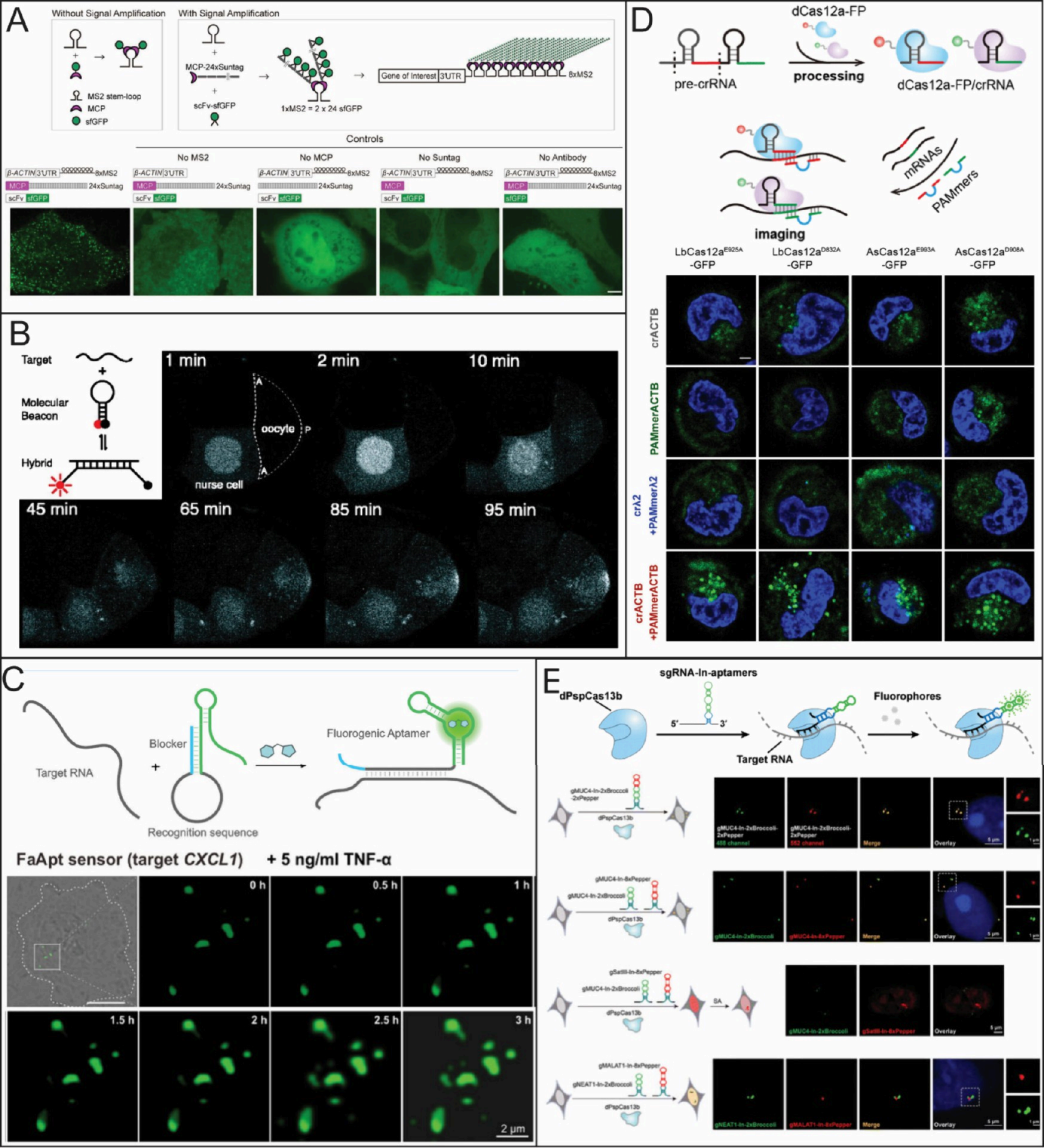
Common live-cell imaging techniques used for RNA quantification. (A) Schematic of MS2-MCP system and MS2-based signal amplification with the suntag system (top) and representative live-cell images of β-actin (bottom). Reporduced from ref 67 under Creative Commons License CC BY 4.0. Copyright 2023 Hu Y.; et al. Published by *eLife*; (B) Illustration of Molecular beacons for live-cell imaging being used to visualize the transport of native oskar mRNA from a nurse cell to the posterior cortex of the oocyte. Adapted with permission from ref 82. Copyright (2003) National Academy of Sciences, U.S.A. (C) Example of fluorogenic RNA being used to target CXCL1 mRNA after 5 ng/mL TNF-α treatment. Adapted with permission from ref 68. Copyright 2023 American Chemical Society. (D) Example of different dCas12a mutants fused with GFP in the presence of a PAMmer sequence targeting β-actin mRNA in HeLa cells. Reporduced with permission from ref 77. Copyright 2024 American Chemical Society. (E) dCas 13b with different RNA sgRNA aptamers for multicolor imaging of *MUC4* and *SatIII* RNA. Reproduced from ref 79 under the Creative Commons License CC BY-NC 3.0. Copyright 2022 Tang, H.; et al. Published by Royal Society of Chemistry.

#### 3.2.2 Fluorogenic RNAs

Fluorogenic RNAs are genetically encoded RNAs that form a stable secondary structure and bind to fluorescent dyes. Instead of expressing fluorescent proteins that bind to the RNA secondary structure like the MS2-MCP system, membrane permeable fluorescent dyes bind to the RNA and emit light to generate the RNA-specific signal (Figure 2C).^69^ Kaiyi Huang et al. developed the fluorogenic RNA named Pepper that binds to derivatives of a synthetic dye HBC. HBC is clear in solution and fluoresces upon binding to pepper.^70^ Compared to the MS2-MCP system, the size of the fused fluorogenic RNA (49 nt) is much smaller than both the 24x and 8x stem-loop RNA sequences mentioned above. In 2025, the same group developed a fluorogenic RNA that hybridizes to the target sequence instead of fusing to it, which originally was not possible on its own.^71^ This fills the need for methods that can image native, unmodified RNA. This sequence-activated fluorescent RNA (SaFR) undergoes a shape change upon binding to its target sequence. This shape change is necessary for the binding of HBC and fluorescence of the fluorogenic RNA. This method was used to monitor the assembly and disassembly of stress granules in real time. In addition to monitoring RNA without being fused to a gene of interest, the authors claim that this is the first time that a fluorogenic RNA has been capable of fixed-cell imaging. Other benefits of the technique include its increased sensitivity to low expressed RNA, confident avoidance of interference with RNA functionality, and lower background fluorescence compared to molecular beacons and other fluorogenic RNAs.

#### 3.2.3 CRISPR/Cas Systems

Clustered regularly interspaced short palindromic repeats (CRISPR) and their associated endonucleases (Cas) first made their appearance as a tool for gene editing, but over time became widely used in live-cell imaging.^72–74^ CRISPR/Cas systems are composed of CRISPR RNA (referred to as crRNA, sgRNA, or gRNA), which is complementary to a target sequence (DNA or RNA), and a Cas protein that contains endonuclease domains that cleave the target gene or nearby RNA. The specificity of these systems has led to their use in live-cell RNA imaging.^75,76^ The deactivated proteins lose their nuclease activity but can still bind to their target sequence. dCas9 and dCas12 systems bind to DNA but can also bind to single-stranded RNA when externally supplied DNA oligonucleotide (PAMmer) binds to dCas-crRNA complex; thus, they can be used to track the movement of mRNA (Figure 2D).^77,78^ dCas13 and dCsm can bind to target RNA without the need for a PAMmer. A key advantage of CRISPR/Cas systems is that they can be used to monitor the movement of endogenously expressed RNA. Imaging of endogenously expressed RNA using these complexes can be done by tagging either the Cas protein with a fluorescent protein or the single guide RNA (sgRNA) with a fluorogenic aptamer.^76,79^ The sensitivity and specificity of CRISPR/Cas systems is also higher than molecular beacons (mentioned in a later section^80^) due to the protein assisted binding.

Heng Tang et al. pointed out that tagging sgRNA with fluorogenic RNA aptamers may be the better option due multiple reasons and developed a method named CasFAS (CRISPR-dCas13 system with fluorescent RNA aptamers in sgRNA) to study RNA–RNA interactions.^79^ The method uses a dPspCas13b system with modified fluorescent RNA aptamers Broccoli and Pepper (Figure 2E). They mentioned that a labeled Cas protein could still exhibit fluorescence if not bound to its target, but sgRNA was reported to degrade if not bound to its complementary target RNA. This means labeling the sgRNA would decrease any fluorescent signal being detected from nonspecific binding, leading to a higher signal-to-noise ratio. There is also a limited amount of labeled fluorescent proteins that can be used to tag Cas proteins, but there are more fluorogenic RNA aptamers and dye combinations that span across the visible light spectrum.

In 2025, Chenglong Xia et al. took inspiration from smFISH to develop smLiveFISH.^81^ Where smFISH uses multiple fluorescently labeled complementary oligonucleotides to bind onto a target RNA with high specificity and SNR in fixed cells, smLiveFISH uses multiple GFP-labeled CRISPR-Csm complexes to bind to target RNA in live-cells. *NOTCH2* and *MAP1B* mRNA were used to test the technique. With *NOTCH2*, cotranslational translocation of the RNA was monitored, and two populations of *NOTCH2* mRNA were distinguished by their diffusion dynamics. *NOTCH2* mRNA anchored to the endoplasmic reticulum and undergoing translation was shown to be static or to have slow movement in the cell, which leads to the conclusion that the movement of *NOTCH2* transcripts is dependent on its translation. For *MAP1B*, the authors tracked the movement of transcripts toward the edge of the cell in real time. They noticed that the *MAP1B* mRNA moved in a linear fashion. Their results combined with literature showing *MAP1B* mRNA to bind to protein kinesin-1 (a microtubule motor protein), helped them conclude that *MAP1B* transcripts localize to the edge of cells through directional transport on microtubules.

#### 3.2.4 Molecular Beacons

Molecular beacons (MBs) are complementary oligonucleotides with stem-loop structures and contain dye on one end of the sequence and a quencher on the other.^80,82^ Before binding to their target, the stem-loop structure places the dye and quencher in the proximity of one another, which quenches the fluorescent signal. Binding onto the target RNA strand separates the dye and quencher from each other so that fluorescence can be detected. This reduced background thus allows MBs to be used for live-cell imaging. This technique was developed in 2003 by Bratu et al. to monitor the transport of mRNA in living cells (Figure 2B).^82^ The main challenge of MB in live-cell imaging is that endogenous nucleases cleavage of MBs and nonspecific binding of MBs to other RNA also increases the distance between the dye and quencher, leading to false positive signal and increased background. Increased background can also come from MBs being shown to accumulate in the nucleus. Difficulty to accurately deliver MBs to their target limits their quantitative capabilities outside identifying general trends in mRNA localization and movement.^82,83^

### 3.3 Image Analysis

#### 3.3.1 Intensity-Based Analysis

Intensity-based analysis uses the number of photons recorded within the exposure time to estimate the expression levels in fluorescence microscopy.^84^ The photons collected by a detector are converted into intensity per pixel. The simplest form of intensity-based quantification counts the number of pixels above an intensity threshold in the entire image or a region of interest. This method is commonly done using software such as ImageJ (FIJI) or Cell profiler.^85,86^ The properties of the fluorophore, sample, optics, and detector heavily influence intensity measurements (see reviews in refs 84 and 87–89 ). Using fluorophores or standards (beads or phantoms) with a known concentration can allow the intensities to be converted into the RNA copy number.

#### 3.3.2 Point Pattern Analysis

Point pattern analysis using nearest neighbor analysis, Ripley’s K, and correlation functions for characterizing global clustering, which determines if a set of points in an image are randomly dispersed, present a uniform pattern, or clustered. Nearest neighbor analysis starts from point A, finds the closest point B from A, and then calculates the distance between the two. Nearest neighbor distances can be plotted as histograms to describe the frequency at which two different points are within a given distance.^90,91^ Clustered points have a smaller nearest neighbor distance compared with that of randomly dispersed points. Therefore, the difference histograms can be used as an indicator of clustering (see Figures 3B and 4D for examples).^90^

**Figure 3:**
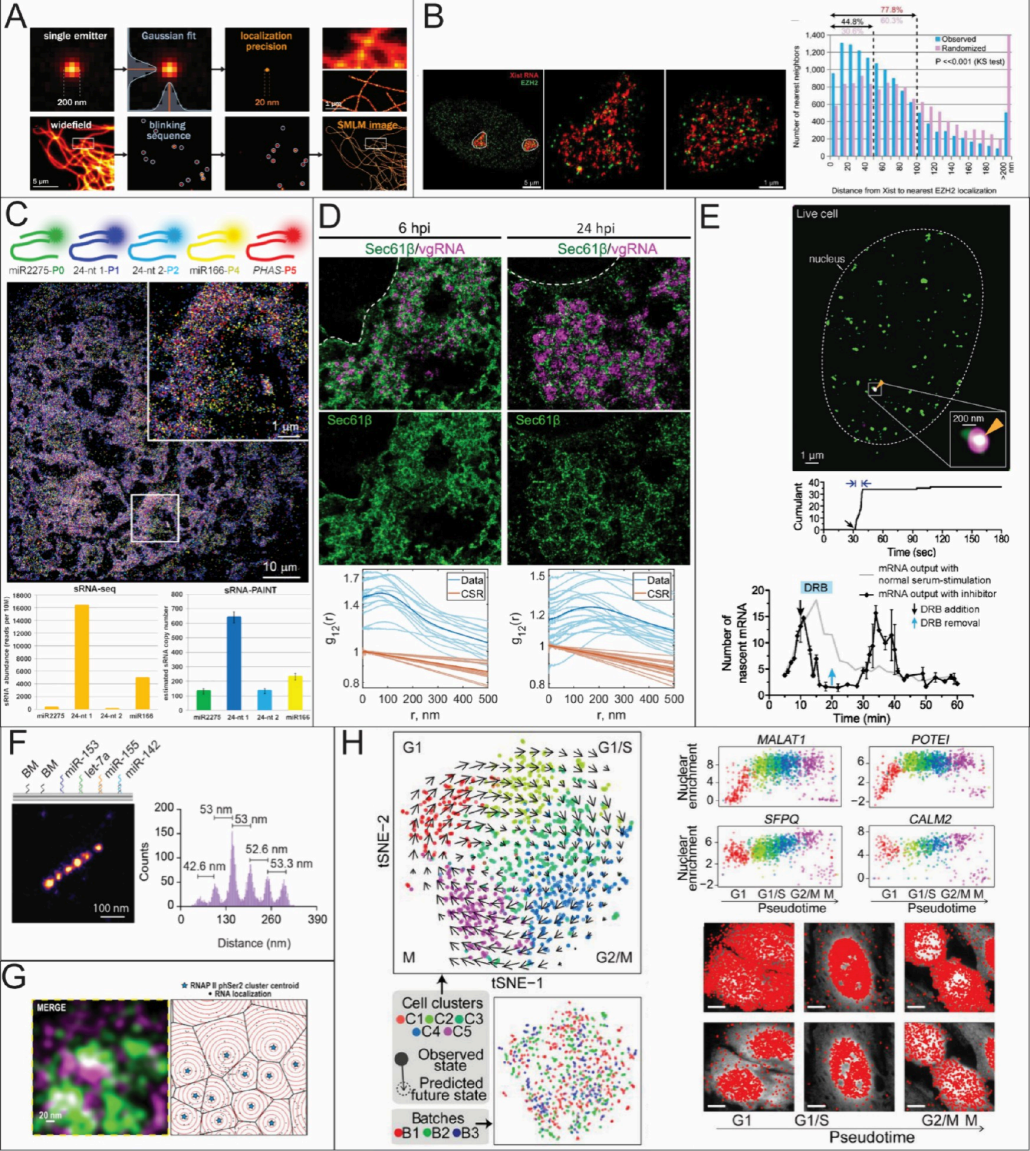
RNA quantification using STORM, PAINT, and ExM. (A) Fluorophore localization for SMLM reconstruction. Reproduced with permission from ref 117. Copyright 2020 Elsevier. (B) Nearest Neighbor distances to count the number of Xist molecules and their distance to a histone marker, respectively. Reproduced with permission from ref 147. Copyright 2015 PNAS. (C) Localization of different sRNAs using sRNA-PAINT and their reported expression levels compared to RNA-seq Reproduced from ref 99 under Creative Commons License CC BY 4.0. Copyright 2020 Published by Oxford Academic Huang, K.; et al. (D) Bivariate pair correlation to measure the correlation between Sec61β with vgRNA and dsRNA and Sec61β with nsp3. Reproduced from ref 115 under Creative Commons License CC BY 4.0. Copyright 2024 Published by Springer Nature. Andronov, L.; et al. (E) Super-resolution time trace of Pol II cluster colocalizing with the active gene locus of β-actin (top) and real-time monitoring of mRNA output of ACTB following serum stimulation (bottom). Reproduced from ref 141 under Creative Commons License CC BY 4.0. Copyright 2016 Published by *elife.* Cho, W.-K.; et al. (F) Detection of miRNA using DNA PAINT. Expression reported by counts and each peak is a different miRNA. Reproduced from ref 13 under Creative Commons License CC-BY-NC-ND. Copyright 2023 Published by Elsevier Kocabey, S.; et al. (G) Voronoi Tessellation of RNA nanodomains clustering to different RNAP II using STORM and DNA-PAINT. Reproduced from ref 149 under Creative Commons License CC BY 4.0. Copyright 2022 Published by Oxford Academic. Castells-Garcia, A et al. (H) Spatial transcriptome wide analysis using expansion microscopy and MERFISH. Reproduced from ref 167 under Creative Commons License CC BY-NC-ND. Copyright 2019 Published by National Academy of Sciences Xia, C.; et al.

**Figure 4:**
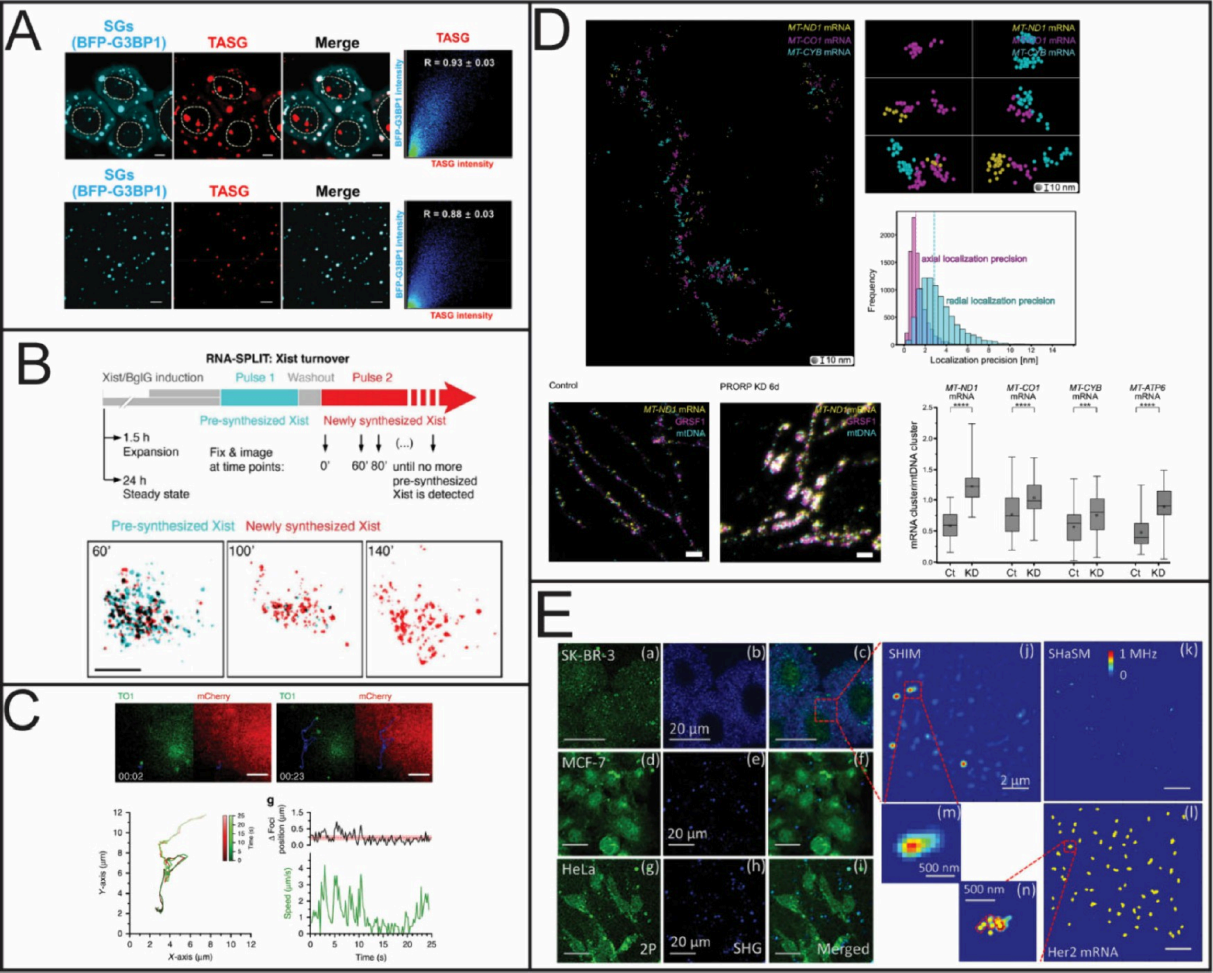
RNA quantification using SIM, STED, MINFLUX, and SHaSM. (A) SIM imaging of stress granules using a small molecule fluorescent probe (scale bar 5 μm). Reproduced from ref 170. Copyright 2023 American Chemical Society. (B) Schematic of using RNA-SPLIT to monitor Xist Turnover and representative 3D SIM images of Xist turnover during expansion. Reproduced with permission from ref 169. Copyright 2021 AAAS. (C) Single particle tracking of the comovement of TOI1-B and tdMCP-mCherry labeled trajectories. Reproduced from ref 171 under Creative Commons License CC BY 4.0. Copyright 2020 Cawte, A. D. et al. Published by Springer Nature. (D) Subcellular characterization of mtRNA using STED and MINFLUX. Reproduced from ref 168 under Creative Commons License CC BY 4.0. Copyright 2025 Stoldt, S.; et al. Published by Springer Nature. (E) Detection of Her2 mRNA in three different cell lines using SHaSM. Reproduced from ref 174. Copyright 2014 American Chemical Society.

Ripley’s K function and derivates like Ripley’s H and L measure the homogeneity of distribution. Ripley’s K function, K­(r), determines if a set of points are randomly dispersed in a given range (distance < r). When plotting K­(r) against r, a clustered sample set will have a higher observed K­(r) at low r and higher observed K­(r) at high r than the complete spatial randomness data set.^92^ Ripley’s L-function L­(r) linearizes the K-function, and Ripley’s H-function H­(r) further normalizes the L-function, so the expected value is 0 when the variation of points is completely spatially random at a given distance. When H­(r) > 0, the points in a sample are considered clustered, and when H­(r) < 0, the points are dispersed.^93,94^ Ripley’s function has been used in diffraction-limited microscopy, with one example being Ripley’s H function to measure the distribution of mRNA in 3D using both wide-field and confocal microscopy.^95^

Correlation functions can be broken into pair correlation and cross correlation.^96,97^ Pair correlation calculates the probability of finding a point at a given distance from another point. Cross correlation is used to measure colocalization for multicolor images and calculates the probability of finding a point in one channel at a given distance from a point in another channel.

#### 3.3.3 Cluster Analysis

The types of analysis described above can give a global representation of the distribution of localizations or clusters of an image. However, other approaches need to be implemented to quantify the different characteristics of individual clusters within an image. This type of quantification is important to characterize clusters of expressed RNA within a cell, which could vary based on a cell-cycle stage, cell differentiation, and disease-related state.

Density-based spatial clustering of applications with noise (DBSCAN) is the most common clustering approach and is useful in identifying clusters of arbitrary shape. DBSCAN identifies a collection of spatial coordinates as unique clusters by defining the minimum number of points (*MinPts*) within a specific radius (ε). Points that are not within the user defined radius are treated as noise and are filtered out of the data set.^98^ As mentioned in section 4.1.2, DNA-PAINT, DBSCAN was used by Huang et al. to cluster images of small RNA (sRNA) acquired with sRNA-PAINT, a version of DNA-PAINT that is optimized to image sRNAs.^99^ Data analysis was performed using the software Clus-Doc, which combines cluster detection and colocalization for single molecule localization microscopy data.^100^ The major limitations of DBSCAN are the parameters having to be set by the user, and the speed of the algorithm declines as the size of a data set increases. Different algorithms were developed to select the ideal ε and *MinPts* for DBSCAN, and the development of a grid-based clustering algorithm was made to increase the speed of clustering large data sets.^101–105^

Tessellation-based clustering using either Delaunay triangulation or Voronoi tessellation can be used to spatially represent clusters. Voronoi tessellation partitions the whole image plane into polygons based on the distribution of points, while Delaunay triangulation picks the nearest points to create triangles that visualize the distribution. Voronoi tessellation uses the Euclidean distance between different points (referred to as sites or seeds) to generate Voronoi cells; each corresponds to a specific seed. The seed can be a single signal point or an unresolvable dense spot. The Voronoi area size distribution of the observed data set can be compared to the complete spatial randomness data set, as clustered samples will have more Voronoi cells of smaller area.^106,107^ As mentioned in section 4.1.1, PALM/STORM, Alvaro Castells-Garcia et al. made either RNAP II phSer2 or nucleosome clutches as the seeds of Voronoi plots and measured the distribution of RNA within each polygon.^149^ The authors performed this analysis using the method ClusterVisu.^108^ The main challenge of applying Voronoi tessellation to super-resolution imaging (especially SMLM described in later sections) rises from the artificial clusters that are generated from multiple photoblinking events or localization errors, and more user defined parameters are needed to generate Voronoi cells compared to DBSCAN. Improvements to Voronoi tessellation by combining it with other methods like DBSCAN to filter out noise, and deep learning algorithms have been developed, making even 3D clustering achievable.^108–110^

Bayesian probability, which interprets the probability as the reasonableness of an expectation, has also been applied to clustering analysis. Recent Bayesian clustering analysis of images uses a variety of algorithms, each proposing a different clustering scheme. Prior knowledge about the distribution of localizations, which can be captured using functions like Ripley’s K, is preferred for Bayesian methods of clustering to be used to score the various clustering schemes based on a Bayesian generative model. The model uses the distribution of molecules, calculated using point pattern analysis, to score the different clustering schemes with the highest scored scheme being the main output of the algorithm. The use of multiple different clustering schemes and having them scored using analyzed point distributions removes the bias of a user entering any parameters like having to input the number of localizations needed per cluster.^111–114^ As mentioned in section 4.1.1, PALM/STORM, Leonid Andronov et al. used a Bayesian Information Criterion-optimized Gaussian Mixture Model (BIC-GMM) to quantify the transformation of viral genomic RNA in SARS-CoV-2 infected cells.^115^

## 4 Super-Resolution Microscopy for RNA Quantification

Conventional diffraction limited fluorescence microscopy has a resolution limit of about 200 nm. In optical systems such as fluorescent microscopes, emitting objects appear as blurred point spread functions (PSFs). The resolution of the images depends on how well PSFs can be separated. For widefield and confocal microscopy, the quality of the PSF is determined by the emission wavelength of the fluorophore and the numerical aperture of the objective lens used. This means that at best the highest resolution that can be achieved by these methods alone is approximately 200 nm, which is known as the diffraction limit. Therefore, molecules that are in the vicinity of another molecule within 200 nm require other methods to be resolved and analyzed.^116^

Super-resolution microscopy consists of an array of methods that generate images of samples at a resolution higher than the diffraction limit, by changing the emission of fluorophores between on and off states to limit overlapping of PSFs, by increasing the physical distance between fluorescently labeled molecules, or by using interference patterns. The methods that correspond to these approaches are single molecule localization microscopy (SMLM),^117^ expansion microscopy (ExM),^118^ stimulated emission depletion (STED),^119^ minimal fluorescence photon fluxes microscopy (MINFLUX),^120^ and structured illumination microscopy (SIM),^121^ respectively. Some techniques of spatial and temporal distribution analysis used in diffraction-limited microscopy can be applied to super-resolution images, but the superior resolution provides evidence that could not be resolved before the development of advanced microscopy.

### 4.1 Single Molecule Localization Microscopy

Single Molecule Localization Microscopy (SMLM) consists of (fluorescence) Photoactivatable localization microscopy ((f)­PALM), stochastic optical reconstruction microscopy (STORM), and DNA points accumulation for imaging in nanoscale topography (DNA-PAINT).^117,122–125^ The shared principle between these methods is that they achieve super-resolution imaging by utilizing appearance, disappearance, or blinking of the fluorophores to distinguish fluorophores that have overlapping point spread functions.^126^ The difference in each SMLM technique lies in how appearance, disappearance, or blinking is achieved for image acquisition. We only discuss the SMLM quantification methods that have been used for RNA quantification in this review, as there are many review articles on how SMLM can generally be used in research and how to choose the best algorithm for the quantification of reconstructed SMLM images.^127–131^ Additionally, the quantitative methods mentioned below will be discussed under the assumption that the SMLM image has already undergone preprocessing steps such as drift correction and the alignment of multichannel images using TetraSpeck beads, DNA origami structures, or simulated images.

The position of each fluorophore is fitted using a Gaussian distribution. The appearance, disappearance, or blinking of fluorophores in SMLM is a stochastic process; therefore, only a portion of fluorophores will emit light during a given recording period (frame time). The center of the Gaussian fitting curve gives the center of the fluorophore, which is a single intensity-free dot with a XYZ coordinate (Figure 3A). The XYZ coordinates of all of the identified fluorophores from different frames are used to reconstruct the image of the original sample. In general, with an EMCCD camera or an equivalent, SMLM methods can confidentially reconstruct images at a resolution up to 20 nm. Various software packages are available for obtaining the localization of each fluorophore and stacking each frame to generate a reconstructed image.^132–134^ The XYZ coordinate fluorophores as intensity-free points can be used for spatial quantification.

There are many features that make SMLM an attractive method for super-resolution imaging and quantification of RNA. SMLM images can be acquired using a wide-field fluorescent microscope, which can be enhanced by using a confocal microscope. Sample preparation and staining procedure are the same as traditional fluorescent microscopy methods, with only differences in the buffer conditions to allow fluorophore bleaching/blinking (for PALM/STORM) or modification in probe design (for DNA-PAINT) to generate the required signal pattern.

The main limitations of SMLM are the requirement of thin samples and expensive data collection and processing. Imaging beyond the diffraction limit and quantifying RNA in thick samples (e.g., tissue) with SMLM is difficult since it would be much harder to detect enough emitted photons to localize and quantify labeled RNAs. However, a recent advance in the technique has the potential to overcome this limitation.^135^ On the data collection side, due to the random nature of blinking, thousands of frames (a single file size is above a few GB) are needed for generating a single image from SMLM. Reconstructing one SMLM image from a few GB with an intermediate-high speed CPU at the time of this review takes at least 10–30 min, depending on the number of color channel and blinking events. Overlapping spots under SMLM can have a major impact in downstream analysis like colocalization and clustering.^136^ The long data collection time, massive data size, and complex data processing are the main reasons that make SMLM less attractive than STED and MINFLUX (see later sections). See Table 1 for a comparison between different super-resolution microscopy.

**Table 1:** Comparison of Different Super-Resolution Microscopy for RNA Quantification

Technique	General Principle	Advantages	Disadvantages	ref
SMLM	Uses blinking principle to locate the center of a dye.	•Similar labeling and sample preparation as most conventional fluorescent microscopy	•Requires thin samples	^99^,^115^,^124^,^126^,^127^,^141^,^147^
		•Multicolor imaging	•Mainly fixed samples	
			•Mainly 2D images	
			•Large data storage and long data processing times	
ExM	Increases distance between molecules through expansion in a hydrogel matrix.	•Thick cell and tissue samples	•Only fixed cells	^62^,^118^
		•Multicolor imaging		
		•3D imaging		
		•Easy to combine with another microscopy		
STED	Uses depletion laser	•1–10 nm resolution	•Not many reports on RNA imaging	^168^,^184^
		•3D imaging	•Expensive high-power lasers	
			•Strong lasers cause fast photobleaching	
MINFLUX	Uses differential emission intensity of dyes with doughnut shape laser and unique scanning pattern.	•Low photon budget (compared to SMLM)	•Complex instrument alignment	^168^
		•3D imaging	•Long acquisition time for static images	
		•Multicolor imaging	•Small field of view	
		•1–10 nm resolution		
SIM	Uses patterned illumination and reconstruction from images at different phases shifts and rotations	•Small raw data size (9–15 images).	•Complex instrument alignment	^121^,^169^,^170^,^185^
		•Least invasive for live-cell imaging	•Lowest resolution	
		•3D imaging		

#### 4.1.1 PALM/STORM

The shared principle between PALM and STORM is that they achieve a resolution beyond the diffraction limit by having fluorophores switch between an “on” and “off” state at random through unique dye chemistry.^126^ Fluorophores in the on state can emit photons when excited by a laser, while those in the off state cannot. Typically, STORM uses photoswitchable synthetic cyanine dyes that can reversibly switch between on and off states using a thiol- and oxygen scavenger-containing buffer solution.^126–137^ The thiol group reversibly reduces the fluorescent dyes, which switch the dyes from an on state to the off state, while the oxygen scavenger removes oxygen from the system to reduce photobleaching. Each frame in STORM consists of multiple photoswitchable fluorophores being randomly activated and subsequently emitting light when hit with an excitation beam. In contrast, PALM uses photoactivatable, photoconvertible, or photoswitchable proteins to switch from an on to an off state permanently.^138^ A new frame in PALM is not started until all of the excited molecules are photobleached. PALM is the ideal technique for live-cell imaging.^139^ This is in part due to the photoactivatable fluorophores that are compatible with PALM not requiring the cytotoxic buffer conditions that are needed for the synthetic dyes in STORM to optimally photoswitch. STORM has been used to image and quantify RNA in fixed cells and tissues;^140^ there are also examples of STORM imaging being done in live cells to monitor RNA expression.^141^ PALM is rarely used for RNA imaging due to the need of associating fluorescent proteins to target RNA and the lower number of available PALM compatible proteins compared to the plethora of STORM compatible fluorophores.^138–142^ Despite not being used for directly imaging RNA, PALM has been combined with FISH to study RNA packaging and transcription^143,144^ and PALM has been combined with STORM to understand protein–RNA interactions.^145,146^

##### Gene Regulation

A study in 2015 demonstrated how STORM could be used to gain a better understanding on how the lncRNA X-inactive specific transcript (Xist) silences the gene expression of one of the X-chromosomes in female mammal X-chromosome inactivation (XCI).^147^ STORM was able to provide a stoichiometric relationship of Xist through RNA copy number quantification by counting the number of emitted fluorophores. They were also able to determine the colocalization of Xist to different chromatin factors that are reported to directly interact with Xist by quantifying their nearest neighbor distances.

Xist is reported to coat the inactive X-chromosome (Xi) and recruit a polycomb repressive complex 2 (PRC2), which enables the epigenetic deposit of a repressive H3K27me3 mark.^148^ A combination of epigenetic tools (CHART-seq and ChIP-seq) and conventional microscopy led to the illusion that Xist and PRC2 coat the Xi. CHART-seq and ChIP-seq showed the enrichment of Xist across the entire Xi. However, these are ensemble methods that look at multiple cells in a single experiment; therefore, any information regarding Xist localizations at the single-cell and spatial level is lost. When conventional microscopy was used to image Xist at the single-cell level, Xist appeared as a large micrometer-scale amorphous cloud. Sunwoo et al. used two-color and 3D STORM to spatially quantify Xist in individual cells, building upon results from earlier techniques to propose a more refined model on how Xist and PRC2 are involved in X-chromosome inactivation.^147^ Using 3D STORM, the Xist clouds present in conventional microscopy were resolved as different punctum. Each resolved puncta represented approximately 2 Xist molecules and was quantified by counting the number of blinking events, which revealed that approximately 50–100 Xist molecules covered the Xi in MEF cells, which would only be enough to cover 1% of the Xi. Two-color STORM and nearest neighbor distance quantification revealed that Xist and PRC2 puncta were closely associated, but nonoverlapping, which was not achievable previously (Figure 3B).

A 2016 paper by Won-Ki Cho et al. explored the correlation between RNA polymerase II clusters and the expression of β-actin mRNA using PALM and STORM in mouse embryonic fibroblast.^141^ This study introduced an innovative method for live-cell quantification of transcription dynamics at the single molecule level. This was demonstrated by correlating RNA polymerase II cluster lifetime to mRNA output of β-actin and showing that the modulation of cluster dynamics can predictably control gene expression. RNA polymerase II was fused with Dendra2, a photoconvertible fluorescent protein. Β-actin mRNA was labeled in live-cells using the MS2-MCP system. A HaloTag was fused to the MCP protein, and Janelia Fluor 646 (JF646), a far-red organic dye, can covalently bind to the fusion protein. smFISH was used to image β-actin mRNA in fixed cells. PALM was used to image RNA polymerase II and STORM was used for the quantification of mRNA in fixed and live cells.

The number of β-actin mRNA at different gene loci was estimated by comparing the JF646 intensity at the transcription focus to the intensity of diffusing mRNA. The location and abundance of β-actin mRNA signals at four different gene loci were observed. Time-related experiments that were conducted to determine the correlation of mRNA transcription to RNA polymerase II cluster lifetime following serum stimulation to provide further information on transcription kinetics (Figure 3E). The synthesis burst of β-actin mRNA peaked approximately 15 min after serum stimulation. When comparing the transcription of the β-actin gene with RNA polymerase II cluster lifetime, a linear correlation was observed between the cluster lifetime to the number of transcribed nascent mRNA during the first 30 min after serum stimulation. The authors also report a delay of about 2.5 min between peak cluster lifetime and peak mRNA output. This study introduced a generalizable method of using super-resolution to study molecular processes in vivo and capture transient dynamics unachievable with conventional imaging techniques.

##### Genome Architecture

In 2022, Alvaro Castells-Garcia et al. used STORM to measure the relationship between nucleosome clutches, RNAP II and nascent RNA and quantify the amount of local nascent RNA during transcriptional activation.^149^ Euchromatin contains transcriptionally active clutches, wherein the nucleosomes are far enough apart to allow for exposed genes to be transcribed by RNA polymerase II (RNAP II).^150^ These transcriptionally active clutches contain transcription factories that consist of RNAP II, RNA, and other molecules involved in transcription and mRNA processing. The size of these factories can range from approximately 40 to 170 nm in diameter, which is beyond the resolution limit of conventional microscopy.

In this 2022 report, metabolic labeling was used to visualize RNA. Cell media containing 5-ethynyl-uridine (EU), an analogue to uridine that contains an alkyne group, was used with the intention of EU to be incorporated by RNA polymerase. The alkyne group present in EU allowed for click chemistry with a STORM compatible dye (AF647), providing a novel way of using STORM to visualize the distribution and density of nascent RNA in the nucleus.

Voronoi diagrams and nearest neighbor distances were used to analyze the clusters of RNAP II and H2B proteins to nascent RNA. The center of the protein clusters was used as seeds to construct the Voronoi polygons, and circles centered at the seed were generated with 10 nm diameter increment (Figure 3G). In each Voronoi cell, the number of RNA spots distributed within the area between two circles was then measured to give the RNA density as a function of distance from the RNAP II cluster. This analysis revealed that nascent RNAs are organized in structures known as RNA nanodomains. The overall result allowed the authors to hypothesize that the increased distance between H2B and RNA can be from transcription machinery being between H2B and RNA, or from the high compaction and low accessibility to RNAP II at the center of the clutch. This provided key knowledge to better understand the landscape of the genome which is important for the expression of genes and the development of organisms from genetic material.

##### Cellular Environment During Viral Infection

In 2024, Andronov et al. used STORM to quantify SARS-CoV-2 viral RNA and proteins involved in viral replication at early and late stages of infection.^115^ SARS-CoV-2 is a positive sense single-stranded RNA virus; therefore, upon entry of a cell, the RNA can be immediately translated by ribosomes. Electron microscopy (EM) visualized a cell infected with SARS-CoV-2 and showed the existence of double membraned vesicles (DMVs),^151^ which harbor viral genomic RNA (vgRNA) and dsRNA which contains the complementary strand of the vgRNA. Even though EM revealed the shape of DMVs, where vgRNA and dsRNA are located and how they organize inside DMVs could not be confirmed using EM because there is no specific contrast available. Conventional fluorescent microscopy and RNA FISH show vgRNA in low and high levels of infections but were blurred clouds, so confirmation of the spatial relationship between the RNAs, and DMV at different levels of infection needs to be determined using super-resolution microscopy.

Using STORM, more precise observations on the organization of vgRNA at low and high levels of SARS-CoV-2 infection can be made. At low levels of infection, 6 h postinfection in this study, vgRNA clustered in a roundish shape with a diameter of 100–250 nm. At a higher level of infections (24 h postinfection), vgRNA localized in a dense perinuclear network of round shapes with a diameter of 300–700 nm. This change in vgRNA localization was quantified by using a Bayesian Information Criterion-optimized Gaussian Mixture Model. Pair-pair correlation analysis was used to quantify the colocalization between vgRNA and other molecules involved in SARS-CoV-2 replication (dsRNA, replicase related proteins, sec61, and nsp3) at 6- and 24 h post infection (hpi) (Figure 3D). The colocalization analysis of vgRNA and dsRNA revealed that the colocalization of vgRNA and dsRNA changes with the level of infection, as vgRNA and dsRNA are positively correlated with each other at low levels of infection and anticorrelated at high levels of infection. The simultaneous analysis of vgRNA and dsRNA also showed that dsRNA and vgRNA signals were positively correlated only at low levels of infection.

These results led to the conclusion that the generation of negative sense RNA (template used to synthesize vgRNA) decreased at late stages of infection of the cell, which was represented as 24 hpi in their study. Similarly, the amount of replicase proteins did not increase significantly over the course of infection, but the amount of vgRNA did, meaning that the growth of vgRNA comes from a constant amount of replicase proteins. ER membrane protein Sec61 and SARS-CoV-2 nsp3 are involved in the formation of replication organelles (like DMVs) and colocalization using STORM between these proteins and vgRNA confirmed that vgRNA growth occurs in these regions by showing that vgRNA is encapsulated by these proteins.

#### 4.1.2 DNA-PAINT

DNA Point Accumulation for Imaging in Nanoscale Topography (DNA-PAINT) is another SMLM technique used for RNA imaging and quantification.^113^ Unlike STORM and PALM, the on and off states for PAINT originate from the transient binding of freely diffusing dyes or dye-labeled ligands. DNA-PAINT utilizes fluorescently labeled DNA primers (imager strands) that transiently bind to their complementary strands (docking strands), therefore giving an “on” signal when the primer binds to the target for a detectable period and goes “off” when the primer diffuses away rapidly. The docking strand can be the target DNA or RNA molecules or covalently conjugated to an antibody.^152–154^

PALM and STORM rely on the photons emitted by the fluorophores for quantitative analysis. Photobleaching or photoswitching properties can vary depending on the imaging environment. A unique quantitative tool that came from DNA-PAINT is quantitative PAINT (qPAINT).^155^ qPAINT uses the binding kinetics between the image and docking strands to quantify an image, enabling quantification without relying on the photophysical properties of fluorophores. DNA-PAINT also can image more than 3–4 different targets. The number of targets that can be identified in a sample by PALM and STORM is limited to the number of available and compatible fluorophores for the techniques. For DNA-PAINT, the same dye can be used and only the sequence of the imaging strand needs to be exchanged for multiplexable imaging, a method known as exchange-PAINT.^153^ Exchange-PAINT not only reduces the need for multiple dyes for imaging multiple targets but also reduces the number of lasers needed, which reduces the cost of both dyes and instruments.

##### DNA PAINT as a Biosensor

The sensitivity of DNA-PAINT has prompted researchers to develop approaches to make DNA-PAINT a viable diagnostic tool for the detection of nucleic acid content.^12,13^ The change in expression of an RNA could signify the presence of a disease (e.g., cancer, bacterial infection, and viral infection), and the sensitivity of diagnostic techniques could enable earlier detection of the disease so treatments can be administered in a timely manner. RT-qPCR is the current golden standard for detecting disease-relevant nucleic acids.^14–16^ However, there have been multiple reports of RT-PCR being susceptible to generating false positive or negative results based on the efficiency of the PCR phase.^156–159^ The lower stability of RNA compared to DNA increases the probability of RNA degradation during the RT-qPCR process, and the length of smaller RNAs decreases the feasibility of RT-qPCR, since the length of miRNAs is less than or equal to that of the typical primer length used. DNA-PAINT has been used as a PCR-free approach for detecting nucleic acids, as the expression of RNA can be quantified using the fluorescence intensity from multiple blinking events. Not requiring PCR shortens the time of detection, and the accuracy of detection is not dependent on the efficiency of the polymerase.

Zhong et al. combined DNA-PAINT with a CRISPR/Cas13a system based on a dumbbell-shaped hairpin for the detection of viral RNA at attomolar (aM) concentrations.^12^ Hantaan virus RNA (HTNV-RNA) was used to verify their biosensor, and SARS-CoV-2 RNA was used to show the versatility of their approach. DNA-PAINT alone is not suitable for early viral detection due to the low copy number of HTNV-RNA. The authors successfully increased the sensitivity of DNA-PAINT by combining it with the CRISPR/cas13a system and a dumbbell-shaped hairpin (DCP-platform). When binding to a crRNA,Cas13a has RNase activity for both cis cleavage of target ssRNA and trans cleavage of other ssRNA nonspecifically, which has been used as a biosensor of various diagnostic technologies.^160^

The first part of the DCP-platform reported by Zhong et al. consists of a HTNV-RNA-specific crRNA, Cas13a endonuclease, a dumbbell-shaped hairpin DNA, and fluorescent DNA probe that is complementary to the central sequence of the dumbbell-shaped hairpin. The binding of HTNV-RNA to the Cas13a/crRNA complex activates the trans-cleavage activity of Cas13a, which preferentially cleaves the loop regions of the dumbbell-shaped hairpin at a rUrU sequence. Cleavage by Cas13a breaks the hairpin, which exposes the stem region as a primer that anneals the fluorescent probe. The fluorescent probe contains a biotin at the 5′ end, a Cy5 dye at the 3′ end, and a specific cleavage sequence for DNA nucleic acid endonuclease cleavage in the middle. The newly released primer from the hairpin forms a DNA duplex with the fluorescent probe, which signals an endonuclease to cleave the fluorescent probe. The fragments of the fluorescent probe separate and are released from the primer. The unbound primer can again bind to another fluorescent probe to cause cleavage, which produces a significant amount of ssDNA from one primer. The second phase of the DCP-platform introduces streptavidin-coated magnetic beads to remove intact fluorescent probes and ssDNA that contained biotin. The ssDNA with the 5′ Cy5 dye was retained in the supernatant and captured by the ssDNA immobilized onto a coverslip through an 8 nt complementary sequence. The short complementary base pairing allows the transient binding-unbinding needed for DNA-PAINT and acquiring the fluorescent output over 1000 frames generated a high enough signal for detecting a low concentration of viral RNA. The authors reported that the DCP-platform can be completed in an hour.

Kocabey et al. developed another PCR-free method to detect multiple miRNAs at high specificity and low femtomolar (fM) concentrations using a DNA origami nanoarray system with DNA-PAINT.^13^ This method was developed to provide a sensor that could be used for diagnosing different diseases such as cancer and autoimmune diseases. miRNAs are 21–24 nt long ssRNA that play an important role in post-transcriptional gene regulation. This is primarily done by miRNAs binding on the 3′ UTR of mRNA to either promote mRNA degradation or block mRNA translation. miRNA can be secreted to neighboring cells through extracellular vesicles for gene regulation and has been reported to be found in bodily fluids, making miRNAs a promising biomarker for diagnosis of different diseases.^161,162^ Earlier studies using qRT-PCR and RNA sequencing revealed aberrant miRNA expression in different types of disease, particularly cancer.^163^ However, the small size of miRNA and the high sequence homology of some miRNA make PCR probe design challenging, and the false positive result rises during multiple PCR cycles. Analyzing miRNA from body fluids such as blood plasma can be more difficult using PCR and sequencing methods because the concentration of miRNA in body fluids is much lower than in cells.^164^

The authors developed a sensor that can detect the low levels of miRNA located in bodily fluids that could not be detected by using PCR and sequencing. miRNAs from the plasma of patients with early stage breast cancer were isolated, and their sensor was used to quantify the expression profiles of each miRNA. This sensor could differentiate between miRNA sequences that have single nucleotide differences and was used to detect both intracellular miRNA and miRNA in plasma of cancer cells. The sensor is composed of eight DNA double helices that are packaged into a 4 × 2 square lattice and anchor strands protruding out from the top layer DNA. The anchor strands only bind to half of the target miRNA, and the unbound half of the target miRNA can then hybridize to a bridge oligonucleotide which contains a single stranded 8 nt docking sequence for DNA-PAINT imaging. The sensor contained 4 different anchors that were arranged linearly and 53.3 nm apart, and each anchor was specific to miRNA targets. The anchors used had sequences that were complementary to miRNA targets that are overexpressed in patients with breast cancer. The super-resolution imaging by DNA-PAINT can distinguish a bound miRNA target using the distance between the corresponding anchor and the reference “boundary markers”. The number of spots detected at each anchor position is related to the relative expression of their respective miRNA target (Figure 3F). This technique provided a new method to quantify the expression of miRNA in bodily fluid without the need for amplification.

##### Localization of small RNAs

Knowing where miRNAs are in a cell and what targets they colocalize with can provide insight into which genes they may regulate and how the regulation is occurring. Common methods of labeling RNA such as smFISH or immunolabeling are not ideal methods for imaging miRNAs. smFISH works well for mRNAs and lncRNAs, but miRNAs and other sRNAs are too small for multiple probe binding and have poor signal when using smFISH. Labeling the sRNA with a large protein (antibody) could disrupt the interactions between the sRNA and its target. Huang et al. modified DNA-PAINT to be more compatible for imaging and quantifying small RNAs (sRNA-PAINT),^99^ which provides the opportunity for sRNAs to be imaged and quantified at a super-resolution. To image the sRNA, locked nucleic acids (LNA) were designed to hybridize onto the target sRNA. LNAs are RNA analogues that promote ideal Watson–Crick base pair binding by having the 2′-OH group covalently bound to the 4′- C in the ribose sugar,^165^ which improves the specificity of probes used in hybridization-based RNA analysis.

In this report, paraffin-embedded maize anthers were sectioned at 6 μm and were hybridized with LNA probes and imager strand. sRNA-PAINT produced a nanometer resolution image of tissue samples with multiple cellular layers, which revealed unique abundance and localization of sRNAs in differentiated cells (Figure 3C).

### 4.2 Expansion Microscopy (ExM)

Expansion Microscopy (ExM) isotopically increases the size of a sample, separating the physical distance between molecules.^62^ Using this technique, molecules that originally localize within the same diffraction-limited spot can be resolved. This technique utilizes the natural expansion of polyelectrolyte hydrogel when dialyzed. The cell or tissue sample is stained with specific fluorescent labels for target molecules and embedded in polyelectrolyte hydrogel to cross-link the fluorescent labels to the hydrogel polymer framework. The linked sample is homogenized by treating detergent or enzymes (mainly proteases) to remove the mechanical characteristics of the sample, which left a transparent hydrogel with cross-linked labels. Dialysis in water or diluted solvent allows the hydrogel to expand to the desired size, which can be imaged by a diffraction-limited microscope. A clear disadvantage of this technique is that live-cell imaging cannot be done, but the resolution of ExM is limited only by the expansion capabilities of the hydrogel used. ExM has been combined with other super-resolution techniques for further improvements in in resolution.^166^

In 2016, Fei Chen et al. developed a small molecule linker known as “Label X”, so that RNA is anchored to the hydrogel during the expansion process for ExM.^118^ RNA is anchored to the hydro gel through Label X alkylating N7 of guanine. Label X allowed the authors to combine ExM with RNA FISH techniques (smFISH and HCR FISH), which were named ExFISH, to reveal the nanoscale architecture of Xist and NEAT1 lncRNAs. Expanded samples in ExFISH can be restrained with different probes to image multiple mRNAs (serial multiplexing). The resolution capabilities of ExFISH enabled the precise localization of and counting of different RNAs in thick tissue samples simultaneously, which revealed the spatial distribution of RNAs that were originally in diffraction-limited distances.

#### 4.2.1 Transcriptomic Profiling of RNA

As previously mentioned, a major limitation of MERFISH was that highly dense areas of RNA could not be accurately profiled. This is because the fluorescent signals given off by multiple RNAs would overlap and therefore are undistinguishable from one another. This limitation is circumvented with ExM, since densely populated RNAs can be physically separated through expansion. Wang et al. combined MERFISH and ExM to individually identify and count 129 different RNA species in a high-density library. Compared to nonexpansion samples, expanded samples were able to identify the 129 RNA species, which equated to about 13,000 RNA molecules per cell with nearly 100% detection efficiency. Chenglong Xia et al. combined this approach with cellular structure imaging to quantify the distribution and abundance of RNA for individual cells over time, developing a novel way to determine RNA velocity in situ.^167^ Over 1,300 cells were measured, and five transcriptionally different clusters were identified as being in different stages of the cell-cycle using the expression of cell-cycle marker genes (Figure 3H). RNA velocity, which was defined as the time derivative of gene expression state, was determined by the change in nuclear and cytoplasmic mRNA abundance of a specific gene based on the concept that RNA is first synthesized in the nucleus, then exported from the nucleus, and eventually degraded in the cytoplasm. RNA velocity was used to order cells along a pseudotime axis. The transcriptionally different clusters formed a circular pattern along the pseudotime axis and happened to be ordered in a way that also corresponded to different cell-cycle phases, which complemented their earlier results using cell-cycle-specific gene markers.

### 4.3 Stimulated Emission Depletion (STED)

Stimulated Emission Depletion (STED) depletes the emission of the molecules at a given location. Following the excitation of a fluorophore, a subsequent stimulated emission beam (STED beam), promotes the de-excitation of fluorophores.^119^ This STED beam creates a doughnut-shaped distribution, where the fluorophores at the focal region are kept at an off state, where no emission is present/detected. The focal point of a Gaussian is where the intensity is at its highest, but the intensity is essentially zero for the focal point of the doughnut-shaped distribution. The overlapping of the Gaussian distribution beam from an excited fluorophore with a doughnut-shaped intensity distribution, caused by the STED beam, leads to an image that can be detected depending on the size of a detected fluorescent signal around this “zero” region. STED can be used to image RNA in living cells. However, the high-power laser used limits the number of compatible dyes, due to a higher rate of photobleaching, and may have impacts on native RNA secondary structures and cell viability.

### 4.4 MINFLUX

MINFLUX (minimal fluorescence photon flux microscopy) surpasses the theoretical 10–20 nm resolution limit of SMLM and STED by combining some principles from each. MINFLUX uses excitation beams with a local intensity minimum or zero, usually doughnut shaped beams like the depletion beam in STED. The doughnut shaped excitation beam moves in a predetermined path, and the location of a fluorophore is estimated from the differential emission intensity measured at different positions of excitation, which is determined as the location where the excitation zero overlaps with the fluorophore. This allows measuring position of fluorophores within a resolution of 1–3 nm in 2D or 3D.^120^

#### 4.4.1 STED and MINFLUX to Visualize Mitochondrial mRNA

Mitochondrial mRNA has been understudied due to the diameter of the mitochondria being close to the resolution limit of conventional microscopes. This includes the lack of information related to the localization of mitochondrial mRNA to different mitochondrial nucleoids, regions of the mitochondria, mitochondrial proteins, and other mitochondrial mRNA. Both STED and MINFLUX were used by Stoldt et al. to visualize mitochondrial mRNA dynamics and structure (Figure 4D).^168^ Three-color STED with branched DNA smFISH (STED-smFISH) was used to quantify the spatial distribution of three different mitochondrial transcripts (*MT-NDI, MT-CO3,* and *MT-*CYB) under various conditions.^168^ The pairwise distances between *MT-NDI, MT-CO3,* and *MT-*CYB mRNAs were revealed to have similar distributions, with a median of approximately 200 nm. The reported pairwise distances of the different mitochondrial mRNAs are similar to the distances between the same mitochondrial mRNA species, which suggests the mitochondrial mRNAs originate from the same primary transcript. There was a reported 2.1-fold, 1.4-fold, and 1.3-fold decrease in *MT-NDI, MT-CO1,* and *MT-*CYB respectively upon downregulation of PRORP, the catalytic subunit of mitochondrial RNase P. The authors also reported downregulation of mRNAs upon inhibition of POLRMT, a mitochondrial DNA-directed RNA polymerase, and in patients with a specific tRNA-Glu mutation. In apoptotic cells, STED-smFISH was able to visualize the release of *MT-CO1* outside the mitochondrial membrane and quantified a 65% decrease in the number of the same transcripts confined by the outer membrane. Tightly packed transcripts below 30–40 nm, and the folding of single mRNA transcripts are unable to be resolved using STED, so MINFLUX was used to localize individual mRNA molecules in relation to other mitochondrial RNAs and used to study the individual folds of single mRNA transcripts. The median pairwise distance between different transcripts using MINFLUX was 75–91 nm, which is much lower than what was detected using STED. Though STED-smFISH was effective to provide a broad quantitative view of mRNA distribution and abundance, the single nanometer resolution of MINFLUX provided the shape of closely packed mRNA molecules and their relation at a suborganelle level.

### 4.5 Structured Illumination Microscopy (SIM)

Structured Illumination microscopy (SIM) uses structured light to generate special interference patterns known as moiré patterns or fringes. The moiré fringes arise from superimposing two patterns, in this case, the sample and a stripe patterned illumination (structured light) generated from a rotatable diffraction grating in the laser light path. Setting the diffraction grating at different angles generates stripe patterned light with different phases. Imaging the sample with multiple phases sequentially generates multiple moiré fringes of the sample, which is then analyzed to reconstruct a high-resolution image.^121^ SIM improves the lateral and axial resolution of the image over wide-field or confocal microscopy. Compared to STORM or PLAM, SIM does not require photoactivatable or photoswitchable dyes and acquires only 9–15 images of each field of view (SMLM collects thousands). These advantages make SIM a more flexible and lower-cost super-resolution method for both fixed- and live-cell imaging.

#### 4.5.1 RNA Spreading and Turnover

In a landmark paper of XCI study in 2021, Rodermund et al. used 3D-SIM to resolve the distribution and turnover of Xist RNA (Figure 4B), as well as how these are affected by two related proteins (CIZI and SPEN).^169^ Xist RNA was engineered to be inducible by doxycycline and carries 18 Bgl stem-loops for HaloTag fused BglG labeling in mouse embryonic stem cells (mESCs). 3D-SIM revealed that the Xist focal signal gradually increased within the Xist territory during 1.5–5 h postinduction (expansion phase) and was relatively steady at 24 h postinduction (steady state). The relatively fast data acquisition rate of SIM allows the authors to perform live-cell imaging to determine the movement of distinct Xist foci over 5 min. The authors found that Xist signals were largely static under the fastest achievable frame rate of SIM. This suggests that Xist translocation is more rapid than the detection limit (1 frame per 10 s) and led to the development of RNA-SPLIT (sequential pulse localization imaging over time). In RNA-SPLIT, two HaloTag ligands, diAcFAM and JF-585 were sequentially incubated with the live-cell during continuous expression of the Bgl tagged Xist RNA. The color of ligands identifies the Xist RNA synthesized before (presynthesized) or after (newly synthesized) the buffer wash between the two ligand incubation periods. Using RNA-SPLIT with 3D-SIM to quantify presynthesized and newly synthesized samples, the authors identified that Xist RNA stability increases with XCI progression, while Xist transcription is significantly higher in the expansion phase than the steady state. The location of newly synthesized Xist RNA changed from the center of the Xist territories in the expansion phase to more peripherally at steady state. Nearest neighbor analysis from RNA-SPLIT revealed pre- and newly synthesized Xist RNA has a median distance of 160–180 nm, which is higher than the SD-SIM resolution and Xist RNA estimated diameter, indicating nuclear Xist foci signals were in very close spatial association but Xist RNA was not translocated together. Xist RNA turnover and spreading analyses were also conducted with knockout and mutation experiments of two genes encoding a protein crucial for anchoring Xist RNA in somatic cells (CIZI) and a protein crucial for Xist mediated gene silencing (SPEN). The Xist RNA anchoring role of CIZI was confirmed, while a more complex and multifunction role of SPEN was revealed.

#### 4.5.2 Stress Granule Formation

In one example, W. Shao et al. used SIM to study stress granules (SG) in cells and a living multicellular organism.^170^ SGs form when mRNA and proteins undergo liquid–liquid phase separation, usually under cellular stress conditions such as oxidative or heat stress. Formation of SGs leads to tight packing of RNA and proteins, which would be difficult to optically resolve using conventional microscopy techniques. W. Shao et al. developed a small molecule known as TASG, a derivative of the common RNA fluorescent probe benzothiazole cyanine, that selectively binds with SG (Figure 4A). Using SIM, the authors were able to track the assembly, movement, disassembly, and other dynamic changes in living HeLa cells, *Saccharomyces cerevisiae*, and *Drosophila* intestine tissues before and after heat shock. They were also able to track the *in vivo* formation of SGs in living *C. elegans* by quantifying the degree of colocalization between TASG and SG fluorescent signal using Pearson’s correlation coefficient.

#### 4.5.3 Single-Particle Tracking

SIM can also be used with the previously mentioned fluorogenic aptamers and MS2 tagging system for live-cell imaging and the single particle trafficking of RNA (Figure 4C). In one example, Cawte et al. used SIM and an array of fluorogenic RNA aptamer Mango II^171^ for live-cell imaging and single particle tracking of single coding and noncoding RNAs. mRNA localization was quantified using a calculated polarization index (PI) using the mean centroid for both the RNA localizations and the cell as seen in.^172^ Single particle tracking in live cells was accomplished using the TrackMate ImageJ plugin.^173^ The authors were able to resolve the localization of both coding (β-actin) and noncoding (NEAT-1 v1 RNA) beyond the diffraction limit and supported the existing specific localization patterns.

### 4.6 Super-Resolved Second Harmonic Microscopy (SHaSM)

Second harmonic super-resolution microscopy (SHaSM) is another nonfluorescent method for visualizing and quantifying mRNAs beyond the diffraction limit. SHaSM combines a scanning confocal microscopy platform with second harmonic generation (SHG) microscopy (also called second harmonic imaging microscopy, SHIM).^174^ SHG microscopy utilize second-harmonic light generated from nonlinear crystal materials (SHG materials).^175–177^ When SHG occurs, two photons at the same frequency ω are converted to a single frequency of 2ω by the medium. Instead of the use of fluorescent dyes, this technique visualizes filamentous proteins or samples stained with inorganic crystals or SHG dyes such as potassium titanyl phosphate (KTiOPO4, KTP) or carbohydrate.^175^ These nanocrystals are individually excited by a two-photon laser source. The emission of nanocrystals allows for super-resolution imaging.

Although improved SHG microscopy (FS-SHGM) and super-resolved SHG microscopy (rSHG) has been used to visualized collagenous tissue,^175,178^ the article from Liu et al. in 2014 is currently the only known publication related to using advanced SHG for RNA. They reported using SHaSM to quantify mRNA with single copy sensitivity by using barium titanium oxide (BTO) nanocrystals as probes to detect mRNA that encodes for the human epidermal growth factor 2 (Figure 4E).^174^ They were able to quantify the expression of this mRNA in different cell lines by counting the number of dimers present. The dimers were between the target mRNA sequence and a BTO “monomer” which consisted of a modified BTO nanocrystal that was attached to a thiol-terminated oligonucleotide. It is interesting to note that this method has not been used on RNA since the one publication, which may hint at technical difficulties.

## 5 Nonoptical Microscopy

The main focus of this review was to show the progression of RNA quantification with optical microscopy, but there has also been an emergence in nonoptical microscopy methods for studying RNA structural dynamics.^179–182^ RNA can have complex and unique conformational landscapes and folding pathways. Many methods discussed in this review can be used to study the distribution of RNA or their colocalization with other molecules but cannot determine what conformation the RNA takes upon binding. This missing information is important when trying to understand how RNA interacts with various ligands in a cell, which is of interest in biomedical research.^180^

Atomic force microscopes (AFM) use a cantilever with a molecularly sharp probe at the end to trace the topography of a sample by detecting the force between the probe and sample.^183^ AFM images of RNA were recently combined with unsupervised machine algorithm and deep neural networks to develop a novel method, HORNET, for generating low-resolution 3D topological structures of RNA conformers in solution.^180^ Cryo-electron tomography (Cryo-ET) is another nonoptical microscope technique that has gained traction in being used for RNA analysis.^182^ Combination of these nonoptical microscopies with other RNA quantification methods mentioned in this review may further our understanding of cellular function and responses.

## 6 Conclusion

RNA quantification as a field has rapidly advanced from ensemble-based techniques to high-resolution spatially informed techniques. Although powerful, advanced microscopy cannot completely replace traditional ensemble methods but supplies more information and should be used in combination with other methods. For example, smFISH has been shown to detect viral RNA in patients that tested negative using RT-qPCR, which demonstrates the importance of more sensitive techniques for detecting RNA in the field of diagnostics and therapeutics. However, RT-qPCR has a higher throughput and provides quantitative information in a faster and more cost-efficient manner than most microscopy techniques. Similarly, MERFISH and CRISPR/Cas provide targeted, multiplexed approaches to studying in fixed and live cells, respectively. RNA-seq methods are still required, as they have broader transcriptome coverage, can reliably measure high abundance transcripts, and do not require prior sequence information for transcript identification. sRNA-PAINT provided a novel way to image small RNAs in cells in situ, which was originally unattainable due to their short nucleotide length. Recent imaging strategies, like smLiveFISH or optimized fluorogenic aptamers, can potentially be combined with live-cell-compatible super-resolution techniques to uncover more about RNA movement in a cell. The combination of these techniques to quantify RNA is vital for our understanding of complex cellular dynamics, such as the change in RNA in cells and tissues in response to growth, disease progression, and treatments. The knowledge gained can be used to develop more precise diagnostic and therapeutic strategies.

There are still technical and practical challenges that hinder the distribution of advanced microscopy in the RNA research field. The crowdedness of the cell and the nature of RNA make it hard to precisely localize RNA and robustly distinguish signals from noise. Complex sample preparation protocols cause information loss during the process and human error. Some of these limitations can be overcome by combining ensemble and super-resolution techniques, as suggested above. Some other potential solutions, e.g., improving camera resolution or scanning motor precision, rely on the advancement of information technology and engineering. Using fast-evolving artificial intelligence techniques to support massive data analysis and to make robots for sample preparation can also be potential solutions. Practically, the complexity of the super-resolution microscopy makes instrument setup, operation, and maintenance costly. Commercialized super-resolution microscopes come in a box and with service contracts can reduce the expertise requirements at the application site, e.g., core facilities in university, and the cost may be reduced with advances in engineering, sufficient supply chains, and competition. We expect that the continuous efforts from academic and industry researchers will keep improving the application and widespread use of these advanced techniques.

## +

Department of Chemistry and Biochemistry, Western Kentucky University, 1906 College Heights Blvd., Bowling Green, Kentucky, USA 42101
